# The association between maternal postnatal depressive symptoms and offspring sleep problems in adolescence

**DOI:** 10.1017/S0033291716002427

**Published:** 2016-10-20

**Authors:** A. K. Taylor, E. Netsi, H. O'Mahen, A. Stein, J. Evans, R. M. Pearson

**Affiliations:** 1 Centre for Child and Adolescent Health, School of Social and Community Medicine, University of Bristol, Oakfield House, Oakfield Grove, Bristol BS8 2BN, UK; 2 Department of Psychiatry, University of Oxford, Warneford Lane, Oxford OX3 7JX, UK; 3 Department of Psychology, College of Life and Environmental Sciences, University of Exeter, Exeter EX4 4QG, UK

**Keywords:** Adolescence, Avon Longitudinal Study of Parents and Children, maternal depression, sleep

## Abstract

**Background:**

Sleep problems are associated with increased risk of physical and mental illness. Identifying risk factors is an important method of reducing public health impact. We examined the association between maternal postnatal depression (PND) and offspring adolescent sleep problems.

**Method:**

The sample was derived from Avon Longitudinal Study of Parents and Children (ALSPAC) participants. A sample with complete data across all variables was used, with four outcome variables. A sensitivity analysis imputing for missing data was conducted (*n* = 9633).

**Results:**

PND was associated with increased risk of sleep problems in offspring at ages 16 and 18 years. The most robust effects were sleep problems at 18 years [adjusted odds ratio (OR) for a 1 s.d. increase in PND, 1.26, 95% confidence interval (CI) 1.15–1.39, *p* < 0.001] and waking more often (adjusted OR 1.14, 95% CI 1.05–1.25, *p* = 0.003). This remained after controlling for confounding variables including antenatal depression and early sleep problems in infancy.

**Conclusions:**

PND is associated with adolescent offspring sleep problems. Maternal interventions should consider the child's increased risk. Early sleep screening and interventions could be introduced within this group.

## Introduction

Sleep disturbances and disorders are very common and affect 10–56% of the global population (Stoller, 1994; Chilcott & Shapiro, 1996; Partinen & Hublin, 2005; Leger *et al.* 2008). In adults, experiencing lack of sleep or poor-quality sleep affects concentration, memory and daily functioning in the short and long term (Wolfson & Carskadon, 1998; Gregory *et al.* 2009; Ram *et al.* 2010; Shochat *et al.* 2014), and fatigue commonly occurs alongside insomnia (Hossain & Shapiro, 2002). Sleep problems also affect adolescent school performance as measured by grade point average (Wolfson & Carskadon, 2003; Pagel *et al.* 2007). Shorter sleep duration is associated with common illness, and increases the risk of high blood pressure and other cardiometabolic risk factors in adolescents (Azadbakht *et al.* 2013; Panciencia *et al.* 2013; Orzech *et al.* 2014). This may be linked with evidence showing that sleep-deprived adolescents make less-healthy food choices (Kruger *et al.* 2014). Poor sleep also affects mental health; reduced hours of sleep are associated with potentially severe mental health problems such as depression, anxiety and suicidal ideation (Settineri *et al.* 2012; Wong & Brower, 2012; Sarchiapone *et al.* 2014). Poor sleep quality may co-occur with these disorders or may precede them and be a contributing factor (Wong & Brower, 2012; Sarchiapone *et al.* 2014).

Both Australian and American studies have calculated a high economic burden of insomnia in terms of both direct and indirect costs (National Commission on Sleep Disorders Research, 1993; Hillman *et al.* 2006). Direct costs included costs of healthcare, pharmaceuticals, diagnostic tests and research. Indirect costs included the cost of absenteeism and lost productivity.

Intervening before the development of sleep problems is an important method of reducing their public health impact. Risk factors may include anxiety or depression, low socio-economic status, bereavement, childhood abuse, irregular sleep schedules, cigarette smoking or drug and alcohol misuse (Shapiro *et al.* 1994; Fleming & Shapiro, 1995; Shang *et al.* 2006; Greenfield *et al.* 2011; Jarrin *et al.* 2014). Early life risk factors may present opportunities for early identification and intervention. Early intervention is important because not only are sleep difficulties a problem in their own right, but they may also lead to other problems that have a high public health impact.

Maternal depression is one potential early-life risk factor. It has already been established that maternal depression is associated with offspring sleep problems in infancy (O'Connor *et al.* 2007) and the development of depression at age 18 years (Pearson *et al.* 2013). However, no large cohort studies have addressed the possibility of maternal postnatal depression (PND) being a risk factor for adolescent sleep problems. There are several potential mechanisms for a causal association between early maternal depression and offspring sleep problems: shared genes may increase risk, antenatal depression may have a biological effect on the child while *in utero*, or PND may affect parenting and the development of set sleep/wake routines. However, any observed association may also reflect reverse causality (disturbed sleep patterns in the infants may contribute to depression in their mothers) or confounding by shared vulnerability factors such as depression in the offspring.

Therefore, in the current study we test the hypothesis that maternal postnatal depressive symptoms are associated with an elevated risk of sleep problems in adolescent offspring using Avon Longitudinal Study of Parents and Children (ALSPAC) data. Investigating a potential link will add to existing findings that maternal depression can have lasting effects on offspring development. Bidirectional effects and reporting bias will be considered; these have been difficult to address in previous studies as they have relied on shorter-term follow-ups where timings of maternal depression and infant sleep problems are likely to co-occur and are based on maternal reporting only. The unique strength of this study is that sleep problems at 16 and 18 years old are reported by the child and occur so long after the PND that it is unlikely to be explained by reverse causality. In addition, infancy sleep problems are measured and can be accounted for.

## Method

### Data source

The sample was derived from participants from ALSPAC, which recruited 15 247 pregnant mothers in the early 1990s (Fraser *et al*. 2013) and has followed up the mothers and their offspring since. Ethical approval for the study was obtained from the ALSPAC Law and Ethics Committee and Local Research Ethics Committees, and participants gave informed consent. Further information is available on the ALSPAC study website, which hosts details of all available data through a searchable data dictionary (http://www.bris.ac.uk/alspac/researchers/data-access/data-dictionary/). The current study uses data from children completing questionnaires at ages 16 and 18 years. See Fraser *et al.* (2013) for a full description of this sample. Please note that the study website contains details of all the data that is available through a fully searchable data dictionary (University of Bristol, 2015).

### Sample

Our starting sample was mothers who completed postal questionnaires for PND (*n* = 10 317). A sample with complete data across the exposure, outcome, mediating and confounding variables was primarily used for both exposure variables, i.e. *n* = 2054 for the categorical depression of the first 2 years, and *n* = 2496 for a continuous score for PND. All analyses were repeated post-imputation for missing data in a sample with at least one measure of sleep in the offspring, which enabled prediction of missing sleep data (*n* = 9633).

### Measures

#### Exposure

Symptoms of maternal depression were measured using the Edinburgh Postnatal Depression Scale (EPDS), which is a 10-item self-report questionnaire validated for use in and outside of the perinatal period (Cox *et al*. 1987). Postal questionnaires, including EPDS measures, were administered at approximately 8 weeks and 8 months postnatally, and also when the child reached 1.5 and 2.5 years of age.

EPDS scores greater than 12 have a high specificity and sensitivity in predicting clinically diagnosed depressive disorder. Scoring above 12 on the EPDS on at least two occasions suggests depression that is likely to require treatment (Cox *et al*. 1987).

#### Outcome

Four variables were used to indicate potential sleep problems. At age 16 years sleep outcomes were derived from a self-report sleep diary: sleep problems at age 16 years (yes/no), waking in the night at least once per night at age 16 years, and average time to get to sleep in minutes at age 16 years. At age 18 years sleep problems were measured as part of an interview assessing common mental health disorders (Clinical Interview Schedule-Revised, CIS-R) (Lewis *et al.* 1992) and a binary variable was derived relating to reporting significant sleep problems according to International Classification of Diseases (ICD)-10 criteria or not. Such criteria relate to the duration, frequency and impact of sleep disturbances such as difficulties getting to sleep or waking too often. (See the online Appendix for the full questions.)

#### Confounding variables

The following variables are thought to be associated with both maternal depression and adolescent sleep problems and are important to explore as potential confounding variables or alternative explanations. We considered the following potential confounding variables to be important for the reasons described below:

(1) Depression at age 16 years using the Development and Well-Being Assessment (DAWBA, for outcomes at age 16 years) and the CIS-R (outcomes at age 18 years). This was in order to account for concurrent depression to the sleep assessment, because sleep problems may simply be reflective of depressed mood rather than any independent effect of PND on sleep.
(2) Sleep problems in infancy (6 months, 18 months and 2.5 years) as reported by the mother in order to account for reverse causality (where sleep problems contribute to maternal PND).
(3) Socio-economic factors (maternal education, maternal age at birth, and parity), which may elevate risks of mental health and sleep problems in both mother and child.
(4) Smoking in pregnancy and antenatal depression, in order to account for fetal programming.
(5) We also later controlled for the number of subsequent maternal depressive episodes (number of times the mother is above threshold on the EPDS across 10 occasions up to age 12 years) in the post-imputation sample, in order to assess whether continued exposure to maternal depressive symptoms is important. We did this only in the imputed sample because this variable was only possible to derive in mothers who had completed all 10 EPDS measures and so contained substantial missing data.

### Statistical analysis

Our exposure variable was maternal postnatal depressive symptoms. We conducted analysis using both a continuous score for PND (taken as the average EPDS score from the 8-week and 8-month assessments to give a more stable estimate of symptoms in the first postnatal year) and using a categorical variable indicating persistence of PND. We derived a three-level categorical variable: (1) those never reaching the threshold (non-depressed); (2) those above threshold on one occasion only (transient PND); and (3) those above threshold on the first postnatal EPDS and above threshold at least once in the following three questionnaires (recurrent PND). This is because each analysis provides us with slightly different information. Using the continuous score maximizes statistical power and examines the linear association with population symptoms, whereas the categorical variable allows us to examine clinically relevant groups. This approach led to multiple comparisons and therefore it is important to look at the overall patterns of findings and precise *p* values rather than taking *p* thresholds to determine ‘significant’ effects which would be likely to occur by chance with this many comparisons.

These exposure variables were then regressed on to each of the sleep outcomes described above in separate models. For binary outcomes we used logistic regression models and for continuous outcome measures (duration to get to sleep) we used linear regression models. Regression models were first conducted with just the outcome measures and then, in order to explore the role of different areas of confounding outlined above, we adjusted for these variables with additional covariates in a series of further models. This was important to highlight the importance of these different potential alternative explanations for any observed associations. For example, if the association was attenuated following adjustments for child depression it would suggest that the association is driven by child depression, and only children of depressed mothers who go on to have depression themselves also have sleep problems.

### Imputation for missing data

We imputed for missing data because a complete case analytical approach can lead to biased results if the data are not missing completely at random (Royston & White, 2011). For example, in this study the missing data are likely to follow a systematic pattern relevant to our hypothesis. Those mothers who are depressed are more likely to fail to complete surveys. In addition, offspring with mental health or sleep problems are also less likely to attend research assessments. Therefore, our analysis is likely to be biased towards an underestimate of any association between PND and offspring sleep problems (because we are missing those who have both a mother with PND and a child with sleep problems, i.e. the sample with a positive association between these two variables). However, we can correct for this bias by using available data to predict the values of those with missing data, and we are confident in our ability to build an adequate imputation model for these missing data due to the wealth of auxiliary measures that can be employed for this purpose. For example, given that there is substantial information on sociodemographic variables in ALSPAC that predict missingness, missing information can be assumed dependent on observed data (missing at random assumption). We employed a fully conditional specification as implemented in the multiple imputation (MI) chained algorithm in STATA 13 (StataCorp LP, USA) using all variables described in the analyses and additional sociodemographic indicators of missingness (list available on request) to predict missing data across 100 imputed datasets. Monte Carlo errors were less than 10% of the standard error and fraction of missing information (FMI) values were no larger than 0.8 (Royston & White, 2011). The imputation method is based on regression equations to predict the missing variable. Therefore, the unique associations between each imputed variable and the predictor variables are used and every imputed variable is imputed using a unique set of regression equations. We imputed up to a sample with at least one measure of maternal depression and one infant sleep measure, giving a total of *n* = 9633.

## Results

### Sample

The mean maternal EPDS depression score postnatally was 5.5 (s.d. = 4.4, range 0–27). Of mothers, 10% exceeded the EPDS threshold at 8 weeks, and 6% exceeded the threshold on at least one more occasion in the next 2 years (see Pearson *et al.* 2013 for further description of this sample in relation to PND). Frequencies of sleep problems in offspring of mothers with and without PND are displayed in Table 1. A relatively large proportion of offspring report problems with sleeping (approximately 15% at age 16 years and 22% at age 18 years), and over 40% of adolescents report waking in the night. The frequencies of all indices of problematic sleep were raised in the offspring of depressed mothers (see Table 1).

**Table 1.** Descriptive information *(*based on all available data*)*

	Self-reported sleep problems at age 16 years, *n* (%)	Waking in the night at age 16 years, *n* (%)	Mean time to get to sleep at age 16 years, min (95% CI)	Sleep problems at 18 years, *n* (%)
No PND	445/2985 (14.91)	1139/2776 (41.03)	17.57 (17.05–18.09)	597/2681 (22.27)
Transient PND	13/95 (13.68)	32/86 (37.21)	17.81 (14.76–20.86)	23/78 (29.49)
Recurrent PND	34/169 (20.12)	91/158 (57.59)	19.84 (17.60–22.07)	56/154 (36.36)
Total *n*	3249	3020	3233	2913

CI, Confidence interval; PND, postnatal depression.

### Main effects of maternal PND symptoms on offspring sleep problems

As shown in Table 2, the unadjusted models showed an association between PND and the following offspring sleep problems: taking longer to get to sleep, waking more than once in the night, and sleep problems at both 16 and 18 years. The majority of these findings remained following adjustments for adolescent depression at the time they reported sleep problems, and adjustments for sleep problems in infancy (reducing the likelihood of reverse causality), socio-economic factors and antenatal depression. However, the associations between PND and self-reported sleep problems at 16 years or time to get to sleep were less consistent across models, with little evidence for an association between PND and sleep problems at age 16 years once concurrent depression was accounted for.

**Table 2.** Logistic and linear regression models reporting associations between PND and sleep variables

	Crude complete cases		Adjustment 2: concurrent depression to sleep outcome		Adjustment 3: + sleep problems in infancy		Adjustment 4: + socio-economic factors (education, age, parity)		Adjustment 5: + antenatal depression (average across pregnancy), smoking in pregnancy	
	Persistent PND^a^ (*n* = 2054)	PND score^b^ (*n* = 2496)	Persistent PND	PND score	Persistent PND	PND score	Persistent PND	PND score	Persistent PND	PND score
Self-reported sleep problems at age 16 years	1.41 (0.89–2.24), *p* = 0.142	1.15 (1.03–1.30), *p* = 0.016	1.27 (0.79–2.06), *p* = 0.325	1.12 (0.99–1.26), *p* = 0.081	1.17 (0.72–1.90), *p* = 0.534	1.06 (0.93–1.20), *p* = 0.387	1.17 (0.72–1.90), *p* = 0.534	1.06 (0.93–1.20), *p* = 0.393	1.24 (0.72–2.11), *p* = 0.441	1.07 (0.91–1.27), *p* = 0.415
Waking in the night at 16 years (none or at least once)	1.75 (1.20–2.56), *p* = 0.004	1.17 (1.06–1.28), *p* = 0.001	1.69 (1.14–2.48), *p* = 0.008	1.15 (1.04–1.26), *p* = 0.006	1.66 (1.12–2.45), *p* = 0.011	1.12 (1.01–1.23), *p* = 0.028	1.68 (1.14–2.49), *p* = 0.009	1.12 (1.01–1.24), *p* = 0.025	1.60 (1.04–2.47), *p* = 0.031	1.14 (1.00–1.30), *p* = 0.057
Time to get to sleep at 16 years (in min): coefficient (95% confidence interval)	3.02 (0.35–5.68), *p* = 0.027	0.47 (−0.17 to 1.12), *p* = 0.152	2.78 (0.14–5.43), *p* = 0.039	0.36 (−0.28 to 1.00), *p* = 0.272	2.63 (−0.021 to 5.29), *p* = 0.052	0.22 (−0.44 to 0.87), *p* = 0.518	2.69 (0.036–5.34), *p* = 0.047	0.23 (−0.43 to 0.88), *p* = 0.495	3.10 (0.21–5.99), *p* = 0.036	0.59 (−0.27 to 1.45), *p* = 0.180
Sleep problems at 18 years on the CIS-R	1.99 (1.42–2.80), *p* < 0.001	1.34 (1.23–1.47), *p* < 0.001	1.30 (0.54–2.30), *p* = 0.355	1.21 (1.05–1.40), *p* = 0.008	1.27 (0.73–2.23), *p* = 0.403	1.18 (1.02–1.37), *p* = 0.043	1.28 (0.73–2.24), *p* = 0.393	1.18 (1.02–1.37), *p* = 0.023	1.07 (0.57–1.99), *p* = 0.838	1.25 (1.03–1.52), *p* = 0.021

Data are given as odds ratio (95% confidence interval) unless otherwise indicated.

PND, Postnatal depression; CIS-R, Clinical Interview Schedule-Revised; EPDS, Edinburgh Postnatal Depression Scale.

a Recurrent PND (EPDS score >12 postnatally and again in any following wave) compared with no depression in any of the waves; there were no differences when comparing maternal transient PND with no depression.

b Continuous postnatal depression taken as the average score across the 8-week and 8-month assessments = linear association; odds ratio or coefficient = risk for every five-point increase in EPDS average score.

After imputation for missing data and for the model including all adjustments for confounding variables (shown in Table 3), the associations between PND and offspring sleep problems remained similar, with continued evidence for associations between the two main outcomes found to be associated in complete case data: waking at age 16 years and sleep problems on the CIS-R at age 18 years.

**Table 3.** Results post-imputation

	Linear association with PND score including adjustments for confounding variables, before imputation^a^	Linear association with PND score before imputation, post-imputation for missing data and adjustments for confounding
Self-reported sleep problems at age 16 years	1.07 (0.91–1.27), *p* = 0.415	1.08 (0.96–1.21), *p* = 0.185
Waking in the night at 16 years (none or at least once)	1.14 (1.00–1.30), *p* = 0.057	1.14 (1.05–1.25), *p* = 0.003
Sleep problems at 18 years on the CIS-R	1.25 (1.03–1.52), *p* = 0.027	1.26 (1.15–1.39), *p* < 0.001

Data are given as odds ratio (95% confidence interval).

PND, Postnatal depression; CIS-R, Clinical Interview Schedule-Revised.

a See Table 2.

### Role of later maternal depression

In a final step only possible in the imputed sample with imputed maternal depression at all time points, we were also able to include a final adjustment for the number of further maternal episodes of depression. There was evidence that the inclusion of this variable attenuated the associations between PND and sleep variables, with no evidence for associations with waking at 16 years once these variables are included [odds ratio (OR) 1.08, 95% confidence interval (CI) 0.94–1.23, *p* = 0.272] and weakened evidence for an association with sleep problems at age 18 years (OR 1.17, 95% CI 1.00–1.38, *p* = 0.050). This does not negate the original associations; rather it suggests that some of the association is likely to be explained by repeated exposure.

## Discussion

### Summary of main findings

There was evidence for an association between PND and increased risk of sleep problems across dimensions and at both ages of 16 and 18 years. The most robust effect was on risk of waking at night and sleep problems at age 18 years. There was some evidence that associations with the more subjective reports of sleep problems were partially explained by concurrent adolescent depression because adjusting for depression at the time of assessment diminished associations. However, there was evidence that the association between PND and waking in the night remained after controlling for later offspring depression, infant sleep problems (to account for reverse causality), socio-economic variables and antenatal depression. Of offspring of re-currently depressed mothers, 36% reported sleep problems at 18 years, compared with only 22% of offspring of non-depressed mothers.

Our findings are in line with evidence from a study of adolescent girls, reporting poorer sleep quality (measured subjectively) in girls whose mothers experienced at least two episodes of depression during their daughter's lifetime compared with controls (Chen *et al.* 2012).

### Mechanisms

The mechanisms to explain the associations are unclear from the current data alone; however, some speculation is possible. There are a number of mechanisms that could contribute to the association between maternal PND and offspring sleep problems. These include genetic vulnerabilities shared between mother and child (Goodman & Gotlib, 1999). These shared genetic factors which affect sleep/wake patterns would probably manifest during infancy and in part could explain this association. We have, however, attempted to address this by controlling for infant sleep problems, and there is still a clear association. During the first years of life the sleep/wake pattern will undergo substantial changes and, therefore, any shared genetic sleep vulnerabilities or influences that PND may have on the sleep/wake cycle may not become apparent until the child is an adolescent.

PND is known to affect parenting capacities and may interrupt the development of regulated sleep/wake patterns, first through compromising parenting consistency and sleeping routine and hygiene, which is needed to establish successful sleep patterns. Second, maternal cognitions and processing of information and any perceptions or expectations she may have relating to infant sleep may be affected. For example, parental cognitions about sleep are a known predictor of infant sleep problems particularly as they related to setting limits to parental night-time involvement (Tikotzky & Shaashua, 2012). These in turn could disrupt the infant's development of the ability to self-regulate and thus go back to sleep when they awaken. This may particularly influence sleep as the child develops, as well as the mother–child interaction (Pearson *et al.* 2013). Future studies should investigate the role of potential mediating pathways including such parenting behaviours and cognitions around child sleep. Further mediating pathways could be the child's development of regulation capacities such as executive function or emotional functioning such as depression. Finally, home environment, including noise levels, number of people in the household and changing environments could also mediate associations between maternal depression and offspring sleep (Bartel *et al.* 2015).

A potential explanation of the mechanism for the association between maternal PND and sleep problems may be the chronicity of the offspring's exposure to maternal depression. There was some evidence that that the number of maternal episodes of depression explained at least part of the association between maternal depression.

Bi-directional effects are also important as sleep problems in the early years can act as a major stressor to family life, cause maternal sleep loss and feelings of fatigue and contribute to maternal depressive symptoms. It is therefore possible that early sleep difficulties in the child and maternal depressive symptoms entrain a pattern of bidirectional effects predisposing the child for continued sleep problems and the mother at increased risk of relapses (Stein *et al.* 2014).

Of particular importance may be the timing of maternal depression. There are two proposed mechanisms through which disrupted sleep can affect child development (Sadeh, 2007). First, it may cause daytime sleepiness and reduce alertness. This, in turn, has the potential to disturb behavioural regulation and cognitive functioning. Early childhood is a period during which children learn to regulate their emotions and significant disruptions may affect this process. Second, disrupted rapid eye movement (REM) sleep may reduce the activities required for brain maturation, memory consolidation and learning. During the first 2 years of life the sleep/wake rhythm will undergo substantial developmental changes as a result of both brain maturation and development of the central nervous system. Any disruptions to these processes during this stage may have long-lasting consequences (Sadeh, 2007).

### Strengths and limitations

This is the first study to examine the association between maternal postnatal depressive symptoms and offspring sleep problems in adolescence using a large longitudinal cohort. Child-reported sleep at age 18 years was included, and we were able to adjust for sleep problems in infancy, making reverse causality unlikely. Results following imputation were broadly similar, indicating that the findings could not be explained by imputing for missing data.

However, clinical diagnoses of PND or sleep problems were not made formally by doctors, but inferred from the questionnaires that participants completed (the EPDS only allows screening for depressive symptoms but does not diagnose depressive disorders). Additionally there were no measures of maternal sleep problems that could be controlled for. Surrounding causes of waking in the night (such as physical illness, noise or irregular routine) were not known but would in the future provide important insights into potential pathways. Although there were some missing data, imputing for missing data has reduced the risk of bias. Finally, generalizability of the findings to culturally different populations or those with different sleep patterns may be difficult.

### Implications

Maternal depressive symptoms are a risk factor for offspring sleep problems. Early identification of maternal depression and treatment (with, for example, antidepressants, cognitive–behavioural therapy or interpersonal therapy) of the mother's symptoms could potentially help prevent offspring problems (Howard *et al*. 2014). Interventions for mothers with depression should also consider the child's experience. For example, depressed mothers are increasingly offered support with parenting and it may be important for such approaches to include management of infant sleep. Looking at sleep patterns in the offspring of depressed mothers, potentially in sleep studies, is also important. For example, Symon *et al.* (2012) describe an improvement in infant sleep and maternal wellbeing following an infant sleep intervention focusing on behavioural strategies. Early sleep interventions such as this could be introduced within offspring of depressed mothers, who appear to be a high-risk group according to our findings. Sleep studies may also help identify a specific sleep problem that offspring of depressed mothers may develop, which would guide more specific screening and intervention possibilities.

## Supplementary material

For supplementary material accompanying this paper visit http://dx.doi.org/10.1017/S0033291716002427. click here to view supplementary material
