# Supplementary material for: The association between maternal postnatal depressive symptoms and offspring sleep problems in adolescence
Source: Psychol Med. 2016 Oct 20;47(3):451–9. doi: 10.1017/S0033291716002427 (PMC5244447; doi:10.1017/S0033291716002427)
Supplement: Supplementary file 1 [file S0033291716002427sup001.docx]

**APPENDIX**

A number of questions regarding sleep problems according to ICD-10 criteria were asked as part of the CIS-R:

1. In the past month, have you been having problems with trying to get to sleep or with getting back to sleep if you woke up or were woken up?
2. On how many nights in seven did you have problems with your sleep?
3. Thinking about the night you had the least sleep in the past seven days, how long did you spend trying to get to sleep? Only include time spent lying awake in bed trying to return to sleep. (Options were less than 15 minutes, between 15 minutes and 1 hour, between 1 and 3 hours, and three hours or more.)
4. In the past seven days, how many nights did you spend 3 or more hours trying to get to sleep? (Options were none, between one and three nights, and four nights or more.)
5. In the past seven days, have you woken more than two hours earlier than you needed to and found that you couldn't get back to sleep?
6. What are your sleep difficulties caused by? (Options were noises, shift work or late nights, pain or illness, worries, reason not known or other.)
7. Has sleeping more than usual been a problem for you in the past month?
8. On how many nights in the past seven nights did you have problems with your sleep? (Options were none, between one and three days, and four days or more.)
9. Thinking about the night you slept the longest in the past seven days, how much longer did you sleep compared with how long you normally sleep for? (Options were less than 15 minutes, between 15 minutes and 1 hour, between 1 and 3 hours, and three hours or more.)
10. In the past seven days, on how many nights did you sleep for more than 3 hours longer than usual? (Options were none, between one and three nights, and four nights or more.)
11. How long have you had these problems with your sleep as you have described? (Options were less than 2 weeks, between 2 weeks and 6 months, between 6 months and 1 year, between 1 and 2 years, and two years or more.)
